# 初诊急性早幼粒细胞白血病外周神经浸润1例

**DOI:** 10.3760/cma.j.cn121090-20240705-00253

**Published:** 2024-12

**Authors:** 烨婷 王, 雯 陈, 珂臻 秦, 恒涛 亓, 铁铮 王

**Affiliations:** 山东第一医科大学附属省立医院，济南 250021 Shandong Provincial Hospital of Shandong First Medical University, Jinan 250021, China

患者，男，50岁。1年前被确诊为急性早幼粒细胞白血病（APL），并接受全身化疗，共8个疗程，2023年5月结束化疗，已达到临床治愈标准。2023年7月开始出现左上臂外侧肿痛，腕关节及手指伸直障碍，8月于我院就诊，外周血涂片及骨髓穿刺未见白血病复发征象。脑脊液细胞学检测未见白血病细胞。

2023年8月肌电图提示左上肢拇短展肌及肘以远桡神经支配肌见明显自发电位，均无募集反应。左上肢正中神经腕-掌段运动神经传导速度（MNCV）轻度减慢，于拇短展肌记录，肘部刺激所得复合肌肉动作电位（CMAP）波幅较远端刺激所得降低>50％，左上肢桡神经于上臂处刺激未引出明确CMAP。左上肢桡神经浅支未引出感觉神经动作电位（SNAP），左上肢正中神经SNAP波幅较右上肢降低40％。

肌骨神经超声示左上臂桡神经主干及左前臂正中神经明显增粗（图1），上臂桡神经横截面范围约0.49 cm×0.52 cm，横截面积0.18 cm²，前臂中段正中神经横截面范围约0.68 cm×1.05 cm，横截面积0.36 cm²，增粗的神经回声减低，内部束状结构显示不清，彩色多普勒血流成像（CDFI）内可见点条状血流信号。MRI显示左上臂桡神经主干及前臂正中神经肿大，呈T1等信号，T2高信号改变（图2）。行正中神经及桡神经外科活检进行组织诊断，发现神经肿大，在最厚的部分进行活检，保持神经完整。病理检查显示神经内白血病细胞浸润（图3），免疫组化：CD3（−）、CD20（−）和Ki-67（+）。这一发现证实了正中神经及桡神经白血病浸润。

**图1:**
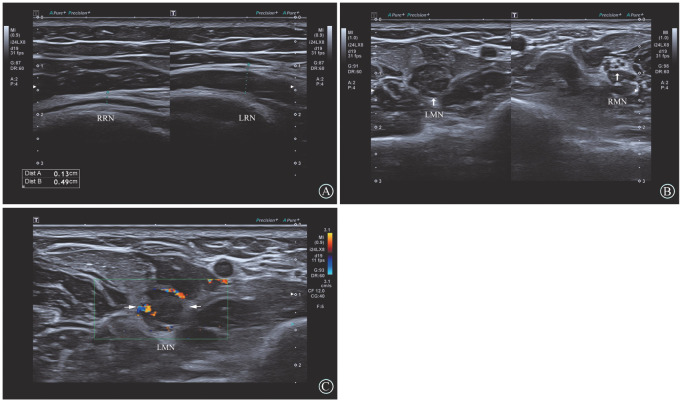
神经超声示左上臂桡神经主干及左前臂正中神经明显增粗，增粗的神经回声减低，内部束膜结构显示不清 **A** 桡神经纵切面的双幅对比图；**B** 正中神经横断面的双幅对比图；**C** CDFI示神经内和神经周围血流信号增多 **注** RRN：右侧桡神经；LRN：左侧桡神经；LMN：左侧正中神经；RMN：右侧正中神经

**图2:**
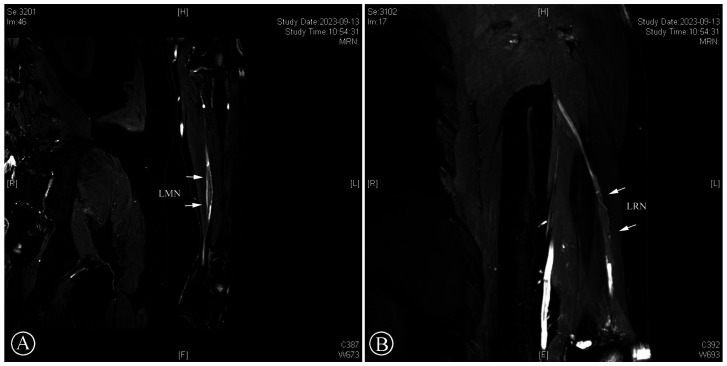
同一患者冠状位MRI图像示正中神经（A）和桡神经（B）病变部位（箭头所示），T2WI呈高信号，神经内部神经束结构受损 **注** LMN：左侧正中神经；LRN：左侧桡神经

**图3:**
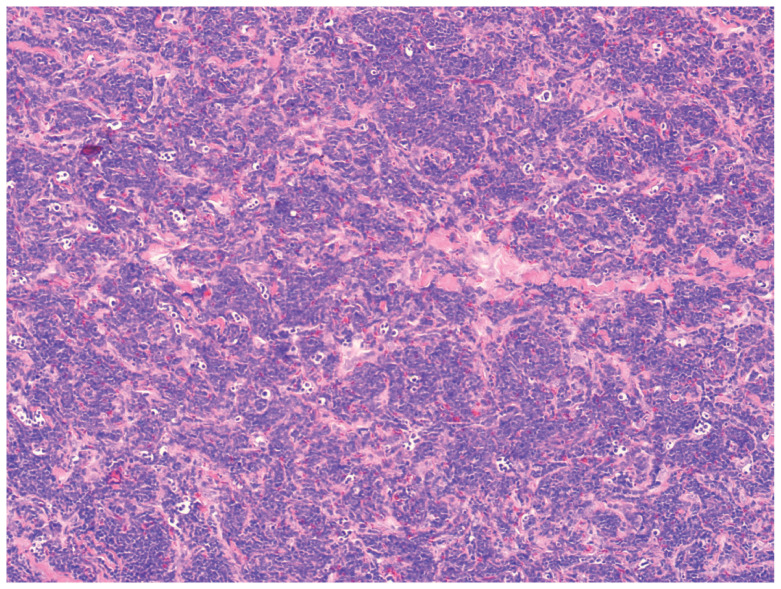
HE染色示神经束膜之间显著的白血病细胞浸润（×100）

**讨论**:神经白血病（NLK）是指白血病浸润周围神经。它通常是由中枢神经系统通过脑脊液或脑膜直接扩散。不累及中枢神经系统的孤立性神经白血病极为罕见。

NLK影像学均表现为受累神经的明显肿胀，束状结构的破坏，增粗的神经内可见有点条状血流信号。白血病细胞浸润神经，破坏神经束膜及神经内膜，对应神经内束状结构的破坏。血流信号的增多与肿瘤相关的血管生成有关。一些研究已经评估了血管生成在白血病外周神经组织内微血管密度和血管内皮生长因子的表达增加，反映在组织病理学亚型的侵袭性上。影像学检查包括超声与磁共振，虽然无法直接定性诊断白血病浸润，但可以准确定位神经病变位置，有助于指导组织取样，同时还可以评估治疗效果。

NLK强烈支持白血病细胞通过血神经屏障（BNB）向周围神经系统(PNS)的血行扩散的概念，而不是周围神经白血病通过神经根的胚细胞纵向播散浸润。白血病细胞可以不受BNB的干扰而进入PNS，而神经内膜内皮细胞之间丰富的细胞间紧密连接使BNB具有限制性屏障的特性，可以阻止全身化疗药物进入PNS。因此，在本例患者中PNS是白血病母细胞的药理学避难所，本例神经活检证实白血病细胞已浸润神经内膜，这与上述理论一致。以往报道的NLK病例大多数在白血病缓解期间出现。本例同样也是被认为临床治愈的白血病患者出现了桡神经及正中神经浸润，外周神经内的白血病细胞的潜伏可能是由于BNB对全身化疗的保护。

NLK发病率极低，非常容易被误诊。主要鉴别诊断为血管性神经病变和格林-巴利综合征（GBS），血管性神经病变表现为不对称多神经病变或多发性单神经炎，而NLK通常表现为单神经病变。此外，与NLK相比，血管性神经病变具有急性到亚急性的表现，后者可能在几个月内缓慢进展。GBS与NLK的临床区别在于弥漫性和对称受累，具有急性/亚急性表现。

NLK被认为是白血病的髓外复发，预后较差。髓内复发的患者需要积极化疗。无血液学复发的患者有较好的短期预后。化疗伴或不伴放疗可显著改善神经系统症状。

